# MEDI3902 Correlates of Protection against Severe Pseudomonas aeruginosa Pneumonia in a Rabbit Acute Pneumonia Model

**DOI:** 10.1128/AAC.02565-17

**Published:** 2018-04-26

**Authors:** Hoan N. Le, Josiane Silva Quetz, Vuvi G. Tran, Vien T. M. Le, Fábio Aguiar-Alves, Marcos G. Pinheiro, Lily Cheng, Li Yu, Bret R. Sellman, Charles K. Stover, Antonio DiGiandomenico, Binh An Diep

**Affiliations:** a Division of HIV, Infectious Diseases, and Global Medicine, Department of Medicine, University of California, San Francisco, San Francisco, California, USA; b Department of Microbial Sciences, MedImmune, LLC, Gaithersburg, Maryland, USA; c Department of Translational Sciences, MedImmune, LLC, Gaithersburg, Maryland, USA; d Pathology Program, Fluminense Federal University, Niterói, Rio de Janeiro, Brazil

**Keywords:** Pseudomonas aeruginosa, therapeutic antibodies, pneumonia, acute lung inflammation, immunotherapy, monoclonal antibodies

## Abstract

Pseudomonas aeruginosa is among the most formidable antibiotic-resistant pathogens and is a leading cause of hospital-associated infections. With dwindling options for antibiotic-resistant infections, a new paradigm for treatment and disease resolution is required. MEDI3902, a bispecific antibody targeting the P. aeruginosa type III secretion (T3S) protein PcrV and Psl exopolysaccharide, was previously shown to mediate potent protective activity in murine infection models. With the current challenges associated with the clinical development of narrow-spectrum agents, robust preclinical efficacy data in multiple animal species are desirable. Here, we sought to develop a rabbit P. aeruginosa acute pneumonia model to further evaluate the activity of MEDI3902 intervention. In the rabbit model of acute pneumonia, prophylaxis with MEDI3902 exhibited potent dose-dependent protection, whereas those receiving control IgG developed fatal hemorrhagic necrotizing pneumonia between 12 and 54 h after infection. Blood biomarkers (e.g., partial pressure of oxygen [pO_2_], partial pressure of carbon dioxide [pCO_2_], base excess, lactate, and creatinine) were grossly deranged for the vast majority of control IgG-treated animals but remained within normal limits for MEDI3902-treated animals. In addition, MEDI3902-treated animals exhibited a profound reduction in P. aeruginosa organ burden and a marked reduction in the expression of proinflammatory mediators from lung tissue, which correlated with reduced lung histopathology. These results confirm that targeting PcrV and Psl via MEDI3902 is a promising candidate for immunotherapy against P. aeruginosa pneumonia.

## INTRODUCTION

The worldwide threat of antibiotic resistance is gradually eroding therapeutic options for the treatment of bacterial infections. In the United States alone, at least 2 million people become infected with resistant bacteria yearly, while 23,000 of these individuals die as a direct result of these infections (1). Globally, deaths attributable to multidrug-resistant pathogens are estimated to reach 10 million by 2050, eclipsing deaths from cancer and diabetes combined (2). This predicament is largely due to overuse and misuse of broad-spectrum chemotherapeutic antimicrobials. Given the continual rise in resistance, the development of new antimicrobial agents is in dire need, including alternative drugs that target single pathogens, such as monoclonal antibodies (MAbs). For Pseudomonas aeruginosa, MAbs targeting either the type III secretion (T3S) protein PcrV or the Psl exopolysaccharide have demonstrated significant potential in preclinical mouse infection models. While anti-PcrV MAbs prevent T3S-mediated injection of exotoxins into host cells (3), anti-Psl MAbs prevent the attachment of bacteria to epithelial cells and promote leukocyte-mediated phagocytosis and killing of P. aeruginosa (4). These distinct MAb mechanisms of action were engineered into MEDI3902, a bispecific bivalent MAb targeting both PcrV and Psl, which conferred enhanced protective activity compared to individual MAbs or a mixture of parental MAbs in mice (5, 6).

In comparison to broad-spectrum antibiotics, pathogen-specific MAbs offer several advantages. This includes preserving the beneficial microbiome while also preventing the spread of resistance in nontargeted microorganisms. MAbs also have considerably longer half-lives than small-molecule antibiotics, enabling extended protection against infection while also making prophylaxis possible. In addition, MAbs generally do not directly kill bacteria and in many circumstances target secreted toxins; consequently, less selective pressure is applied for resistance development. Furthermore, antibody-mediated protection has been repeatedly shown to complement antibiotic therapy in multiple murine models of infection with both Gram-positive and Gram-negative bacteria (5, 7–10). While there are clear advantages for narrow-spectrum antimicrobials, barriers to regulatory approval and commercialization are not trivial. For MAbs against less-prevalent pathogenic species, conducting randomized controlled clinical trials in a cost-effective and timely manner is a significant undertaking (11, 12).

In recognition of the challenges for new pathogen-specific strategies, multiple stakeholders and regulatory agencies have participated in public meetings to explore alternative regulatory pathways to help guide the development of these novel compounds. While no definitive path forward has emerged from these meetings, one pathway under discussion is greater reliance on human pharmacokinetic (PK) data combined with robust preclinical efficacy data derived from multiple animal species (11). To this end, we describe a rabbit acute pneumonia model with a highly pathogenic and cytotoxic P. aeruginosa strain to further evaluate the protective efficacy of MEDI3902 beyond the multiple murine models already tested. We hypothesized that rabbits treated with MEDI3902 will have improved survival outcomes compared to those administered with a control IgG (c-IgG). We further hypothesized that improved survival outcomes will correlate with relevant biomarkers of acute respiratory distress syndrome and remain within normal limits for rabbits receiving MEDI3902.

We found that MEDI3902 significantly protected against acute lung injury, acute lung inflammation, and reduced bacterial tissue burden in comparison to a c-IgG. Analysis of blood biomarkers revealed that many of these parameters were grossly deranged in rabbits receiving control IgG but not in animals receiving MEDI3902. Altogether, these results provide additional support for MEDI3902 as a promising candidate against P. aeruginosa pneumonia.

## RESULTS

### MEDI3902 prevents acute lung injury and lethal lung infection.

**FIG 1:**
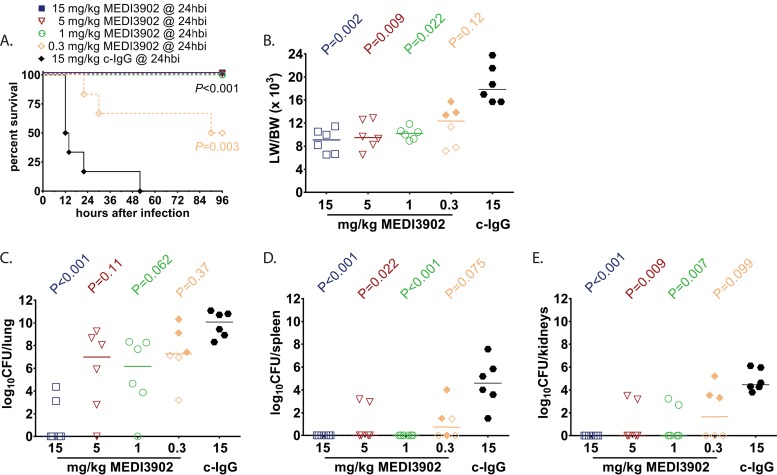
Prophylaxis with MEDI3902 reduces acute lung injury and improves survival outcome. Comparison of Kaplan-Meier survival curves (A), lung weight to body weight (LW/BW ×10^3^) ratio (B), log_10_ CFU/lung (C), log_10_ CFU/spleen (D), and log_10_ CFU/kidneys (E) for rabbits administered intravenously with 15 mg/kg MEDI3902 (*n* = 6) or 15 mg/kg c-IgG (*n* = 6) at 24 h before infection. (B to E) Open symbols represent surviving animals, while closed symbols represent animals that were found dead or were euthanized after becoming moribund and recorded as nonsurviving. hbi, hours before infection. Bars indicate the median for all treatment groups.

Previous work has shown that MEDI3902, a bispecific antibody targeting P. aeruginosa PcrV and Psl exopolysaccharide, mediates potent protective activity in mice (5, 6, 13, 14). Here, we sought to evaluate MEDI3902 activity in a rabbit P. aeruginosa acute pneumonia model while also evaluating its effect on biomarkers that predict disease severity and outcomes. We first characterized MEDI3902 activity in preventing lethal pneumonia in the context of preexposure prophylaxis. Intravenous (i.v.) administration of MEDI3902 to rabbits (*n* = 6/group) 24 h before infection yielded potent concentration-dependent protective activity (Fig. 1A). Animals receiving 15, 5, and 1 mg/kg of body weight exhibited complete protection from lethal acute pneumonia, while 50% of animals receiving 0.3 mg/kg survived infection. In contrast, all animals receiving control IgG became moribund, succumbing to lethal pneumonia between 12 and 54 h after infection (Fig. 1A). Consistent with these survival data, all MEDI3902-treated animals at 15, 5, and 1 mg/kg and surviving animals receiving 0.3 mg/kg at 96 h postinfection presented with reduced lung weight-to-body weight (LW/BW) (×10^3^) ratios, which is a marker of acute lung injury, in comparison with animals treated with control IgG (Fig. 1B). In addition, a significant dose-dependent reduction in bacterial dissemination from the lung to the distal organs was observed only in MEDI3902-treated animals (Fig. 1C to E).

### Effects of MEDI3902-mediated protection in halting acute lung injury and derangement of biomarkers.

**FIG 2:**
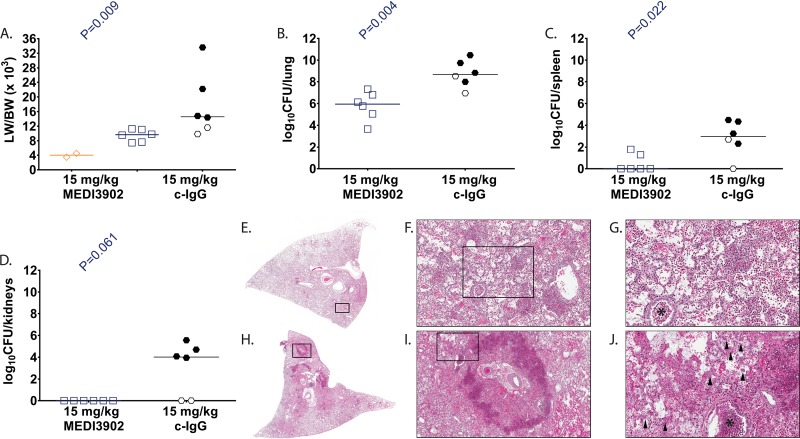
MEDI3902 treatment for evaluation of serum biomarkers and lung histopathology. (A to D) Lung weight-to-body weight (LW/BW [×10^3^]) ratio (A), log_10_ CFU/lung (B), log_10_ CFU/spleen (C), and log_10_ CFU/kidneys (D) for rabbits administered intravenously with 15 mg/kg MEDI3902 (*n* = 6) or 15 mg/kg c-IgG (*n* = 6) 24 h before infection. Four of six c-IgG-treated rabbits died spontaneously from infection between 9 to 10 h postinfection, whereas the remaining rabbits were euthanized at 10 h postinfection. (E and H) Representative gross lung images. (E to J) Hematoxylin & eosin-stained sections of representative lungs from rabbits pretreated with MEDI3902 (E to G) or c-IgG (H to J). Lungs from MEDI3902-pretreated rabbits had moderate infiltrates of viable inflammatory cells admixed with fibrin and edema fluid within alveolar spaces. Bronchioles contain small accumulations of cellular debris (asterisk). Lung from c-IgG-treated animal has multifocal areas of pulmonary necrosis rimmed by consolidated areas of aggregated inflammatory cells admixed with degenerate cellular debris, fibrinous edema, marked hemorrhage, and myriad bacterial colonies (arrowheads). (A to D) Open symbols represent surviving animals, while closed symbols represent animals that were found dead or were euthanized after becoming moribund and recorded as nonsurviving. Bars indicate the median for all treatment groups.

**FIG 3:**
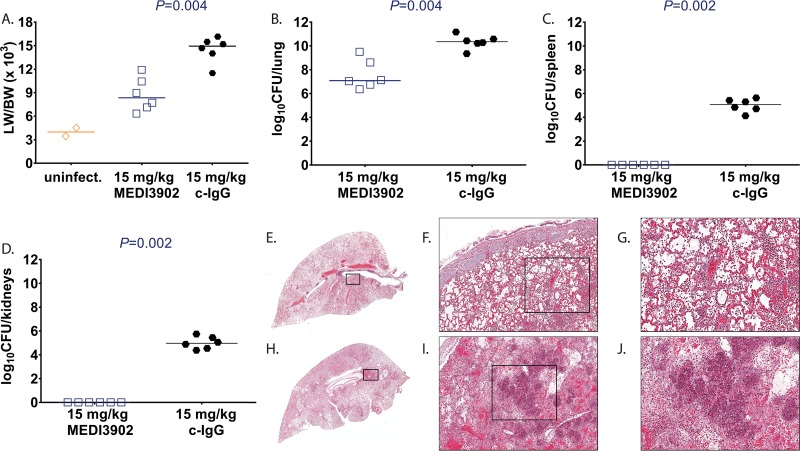
Treatment with MEDI3902 for evaluation of serum biomarkers and lung histopathology. (A to D) Lung weight to body weight (LW/BW [×10^3^]) ratio (A), log_10_ CFU/lung (B), log_10_ CFU/spleen (C), and log_10_ CFU/kidneys (D) for rabbits administered intravenously with 15 mg/kg MEDI3902 (*n* = 6) or 15 mg/kg c-IgG (*n* = 6) 1 h after infection. Two rabbits challenged endobronchially with sterile LRS was included as uninfected (uninfect.) controls. All six c-IgG-treated rabbits died spontaneously of infection between 9 and 10 h postinfection, whereas the remaining rabbits were euthanized at 10 h postinfection. (E and H) Representative gross lung images. (E to J) Hematoxylin & eosin-stained sections of representative lungs from rabbits treated with MEDI3902 (E to G) or c-IgG (H to J). Lungs from MEDI3902-treated rabbits had moderate infiltrates of viable inflammatory cells admixed with fibrin and edema fluid within alveolar spaces. Lungs from c-IgG-treated rabbits demonstrated severe inflammatory with areas of necrosis, hemorrhage, and consolidation. (A to D) Open symbols represent surviving animals, while closed symbols represent animals that were found dead or were euthanized after becoming moribund and recorded as nonsurviving. Bars indicate the median for all treatment groups.

To better characterize MEDI3902-mediated protection in this rabbit P. aeruginosa acute pneumonia model, animals administered MEDI3902 (*n* = 6) or control IgG (*n* = 6) at 24 h before challenge (prophylactic study) or 1 h after challenge (treatment study) were euthanized followed by harvesting of various tissues at 10 h postinfection. Two rabbits were challenged with vehicle control (Lactated Ringer's solution) and served as uninfected controls for treatment (Fig. 2A) and prophylaxis studies (Fig. 3A). In the treatment and prophylaxis studies, 4 of 6 (67%) and 6 of 6 (100%) control IgG rabbits, respectively, became rapidly moribund between 9 and 10 h after infection, whereas all 12 rabbits administered MEDI3902 survived to 10 h postinfection and were euthanized. The 10 control IgG-treated rabbits that succumbed to infection exhibited severe pulmonary edema, with LW/BW (×10^3^) values from 11.5 to 33.6, compared to 3.5 to 4.5 for uninfected control rabbits (Fig. 2A and 3A). The LW/BW ratio (×10^3^) was significantly greater for rabbits administered control IgG than for those administered MEDI3902 (Fig. 2A and 3A). Similarly, bacterial counts for rabbits administered control IgG were significantly greater in the lungs, spleen, and kidneys compared to those administered with MEDI3902 (Fig. 2B to D and 3B to D). In addition, histological analysis showed that lungs from control IgG-pretreated rabbits demonstrated severe neutrophilic inflammatory infiltrates, and it showed multifocal areas of necrosis, hemorrhage, and consolidation (Fig. 2H to J and 3H to J). In contrast, lungs from MEDI3902-pretreated rabbits exhibited moderate infiltrates of viable neutrophilic inflammatory cells mixed with fibrin and edema fluid within alveolar spaces (Fig. 2E to G and 3E to G). Taken together, these data indicate that MEDI3902 protects against severe lung injury resulting from profoundly acute high-level P. aeruginosa infection.

**FIG 4:**
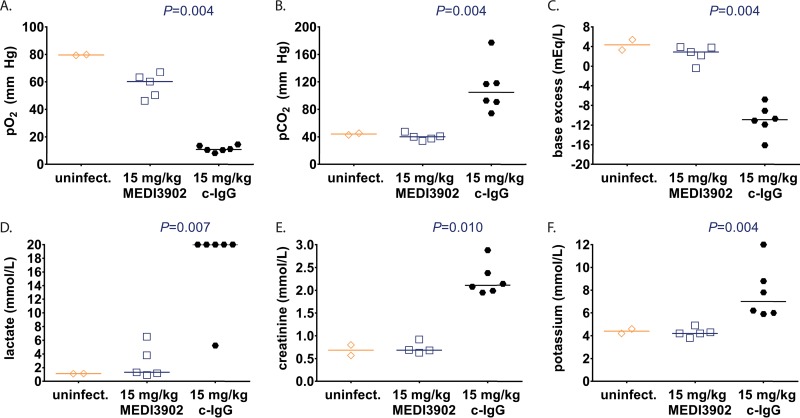
Effects of MEDI3902 on serum biomarkers of disease severity. Comparisons of pO_2_ (A), pCO_2_ (B), base excess (C), lactate (D), creatinine (E), and potassium (F) in terminal blood samples taken from rabbits pretreated with c-IgG or MEDI3902 at 24 h before infection (same animals as shown in Fig. 2). Open symbols represent surviving animals, while closed symbols represent animals that were found dead or were euthanized after becoming moribund and recorded as nonsurviving. Bars indicate the median for all treatment groups.

All six rabbits pretreated with control IgG exhibited profound respiratory failure, as evidenced from extremely low partial pressure of oxygen (pO_2_) (Fig. 4A), high partial pressure of carbon dioxide (pCO_2_) (Fig. 4B), and severe base deficit (acidosis) (Fig. 4C). In contrast, rabbits pretreated with MEDI3902 had decreased pO_2_, but pCO_2_ and base excess levels were within normal limits compared to the uninfected control at 10 h postinfection (Fig. 4A to C). The profoundly impaired gas exchange observed for control IgG-pretreated rabbits resulted in poor tissue oxygenation, as evidenced by extremely high blood levels of lactate that accumulated when cells must generate ATP in the absence of oxygen; in contrast, MEDI3902-pretreated rabbits had significantly reduced blood lactate levels (Fig. 4D). The control IgG-pretreated rabbits also showed evidence of acute kidney injury since they presented with elevated creatinine levels compared to those for MEDI3902-pretreated rabbits (Fig. 4E). In addition, abnormally high potassium levels were evident in control IgG-pretreated rabbits but were within normal limits for MEDI3902-pretreated rabbits (Fig. 4F).

### MEDI3902 reduces acute lung inflammation.

**FIG 5:**
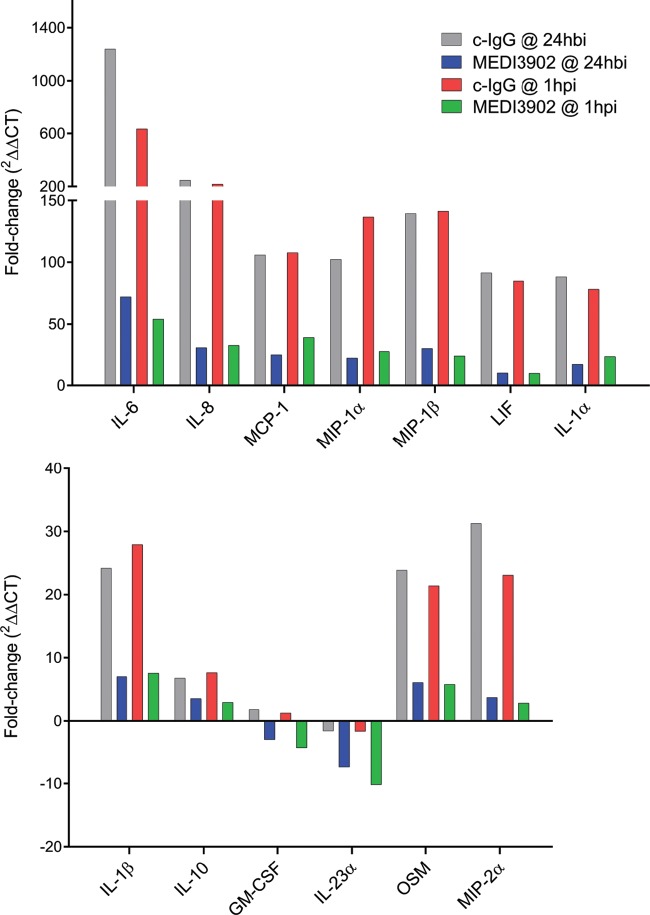
Relative lung expression of inflammatory markers in 6077-infected animals receiving c-IgG or MEDI3902 in comparison to noninfected controls. Antibodies were administered as prophylaxis at 24 h before infection (hbi) (*n* = 6 for c-IgG and MEDI3902) or as treatment 1 h postinfection (hpi) (*n* = 6 for c-IgG and MEDI3902). Noninfected rabbits were treated with normal saline (*n* = 3). Values were calculated from two independent quantitative PCRs (qPCRs)/sample, each using an independent RNA extraction from tissue. *C_T_*s were averaged from animals receiving antibodies for each analyte, and the fold-regulation change was calculated against the average *C_T_*s from noninfected animals.

To characterize acute lung inflammation following P. aeruginosa lung infection in control IgG- and MEDI3902-treated rabbits in both treatment and prophylaxis studies, RNA was extracted from RNA*later*-preserved lung tissue and analyzed by quantitative reverse transcription-PCR (RT-PCR) for changes in expression patterns of inflammatory markers at 10 h postinfection. Compared to the IgG control, MEDI3902 administered either 1 h after (treatment) infection or 24 h before (prophylaxis) significantly reduced the expression of several key proinflammatory markers, including interleukin-6 (IL-6), macrophage inflammatory protein 2-alpha (MIP-2α, or CXCL2), leukemia inhibitory factor (LIF), IL-8, granulocyte-macrophage colony-stimulating factor (GM-CSF), IL-23A, macrophage inflammatory protein-1β (MIP-1β, or CCL4), MIP-1α (CCL3), oncostatin-M (OSM), IL-1α, IL-1β, monocyte chemotactic protein-1 (MCP-1, or CCL2), and the anti-inflammatory cytokine IL-10 (Table 1). A comparison of the fold differences in the expression of cytokines and chemokines from animals receiving c-IgG or MEDI3902 versus noninfected controls (*n* = 3) is presented in Fig. 5. Overall, the reduction in inflammatory gene expression is consistent with MEDI3902 limiting bacterium-induced acute lung hyperinflammation, whereas control IgG is incapable of curbing the explosive acute inflammatory response in the lung, resulting in massive inflammatory infiltrate, tissue necrosis, alveolar edema, hypoxemia, and death.

**TABLE 1:** Effects of MEDI3902 on modulating expression of key inflammatory cytokines and receptors in the rabbit lungs^*a*^

Gene	Treatment (control IgG vs MEDI3902)			Prophylaxis (control IgG vs MEDI3902)		
	Fold regulation	95% confidence interval	*P* value (FDR adjusted)	Fold regulation	95% confidence interval	*P* value (FDR adjusted)
IL-6	−16.4	−53.5, −5.0	0.0023	−12.3	−51.8, −2.9	0.0102
MIP-2α	−9.5	−27.1, −3.3	0.0034	−7.2	−21.5, −2.4	0.0096
LIF	−8.9	−17.4, −4.6	0.0007	−8.7	−19.2, −3.9	0.0032
IL-8	−7.2	−17.5, −3.0	0.0024	−7.7	−20.4, −2.9	0.0075
GM-CSF	−6.7	−15.5, −2.9	0.0024	−4.7	−11.4, −1.9	0.0102
IL-23α	−6.0	−14.1, −2.5	0.0034	−4.3	−12.4, −1.5	0.0283
MIP-1β	−5.9	−15.0, −2.3	0.0079	−4.7	−16.1, −1.4	0.0427
MIP-1α	−4.7	−8.2, −2.7	0.0014	−5.8	−20.7, −1.6	0.035
OSM	−3.9	−7.8, −1.9	0.0084	−3.7	−7.6, −1.8	0.0134
IL-1α	−3.6	−6.6, −1.9	0.0036	−4.9	−10.0, −2.4	0.0072
IL-1β	−3.4	−7.0, −1.7	0.0115	−3.7	−10.4, −1.3	0.0427
MCP-1	−2.7	−5.3, −1.4	0.0154	−4.3	−8.6, −2.2	0.0072
IL-10	−2.5	−4.8, −1.3	0.0232	−2.0	−3.1, −1.3	0.0137

a Real-time PCR was used to analyze the expression of 84 key genes encoding cytokines and their receptors in lungs from rabbits administered c-IgG or MEDI3902 at 24 h before infection or 1 h postinfection. Shown are genes downregulated at least 2-fold due to MEDI3902 intervention in rabbit lungs infected with P. aeruginosa strain 6077.

## DISCUSSION

While no animal model is capable of fully recapitulating human infection, rabbits reproduce many hallmark clinical features of hospital-acquired pneumonia, such as severe hypoxemia, leukopenia/neutropenia, hyperlactatemia, hyperkalemia, and other blood biomarkers due to rapidly progressing infection. In addition, rabbits offer the ability to conduct longitudinal studies for evaluating therapeutic efficacy, including elucidating relationships, such as pharmacokinetics and pharmacodynamics. Moreover, rabbits are phylogenetically more similar to humans than rodents and exhibit similar sensitivities to lipopolysaccharide (LPS), making this species attractive for preclinical and translational research (15–18). Finally, rabbits possess airway anatomy and pathophysiologic responses to pulmonary disease similar to those of humans, eliciting an inflammatory profile in acute bacterial pneumonia of congestion, edema, and neutrophilic infiltrate that parallels infections in humans (16, 19). In this work, we sought to evaluate antipseudomonal MAb MEDI3902, a bispecific antibody targeting the T3S system PcrV protein and the Psl exopolysaccharide, for its ability to inhibit lethal pneumonia in rabbits. Our results confirm the protective effects of MEDI3902 treatment or prophylaxis after endobronchial infection with the cytotoxic and multidrug-resistant (MDR) P. aeruginosa strain 6077. This strain expresses the potent phospholipase exoenzyme U (ExoU) via the T3S system, is acutely cytotoxic to host cells *in vitro*, and causes a high-degree of acute lung injury in murine pneumonia models. P. aeruginosa strains harboring a functional T3S system have been linked to poor clinical outcomes, with ExoU-expressing strains serving as an additional marker for highly virulent infecting strains (20–27).

Whether delivered for prophylaxis or postinfection treatment, MEDI3902 was highly effective in preventing lethal P. aeruginosa acute lung infection. This striking effect on survival correlated with a reduction in bacterial burden in the lung while also preventing the spread of bacteria to distal organs. Consistent with survival and bacterial organ burden data, MEDI3902 was effective in preventing acute lung injury, as evidenced by reduced LW/BW ratios compared to the controls (Fig. 1 to 3). Also noteworthy was the observed preservation of alveolar tissue in our histopathological analyses in MEDI3902-treated rabbits. While lungs from all infected animals showed signs of inflammatory cell infiltrates, only control IgG-treated animals resulted in hemorrhagic necrotizing pneumonia, fibrin-consolidated areas, and observable bacterial colonies (Fig. 2E to J and 3E to J). Besides these effects, MEDI3902-treated rabbits presented normal values of pO_2_, pCO_2_, and minimal acid/base disturbance (Fig. 4A to C). Lactate blood levels, a biomarker of hypoxia and tissue hypoperfusion shown to be useful in evaluating the severity of pneumonia cases (28), also exhibited levels similar to those in noninfected rabbits. The high lactate levels observed in control IgG-treated animals were corroborated by the observed acidosis in prophylaxed animals (Fig. 4C). The lactate levels were similar to those in noninfected control animals in rabbits receiving MEDI3902 and apparently correlate with recent work reporting a potential link between acidosis and increased cytotoxicity due to P. aeruginosa secretion of ExoU (29). In addition, acute kidney injury and cardiovascular dysfunction related to sepsis were also kept in check by MEDI3902, as evidenced by the normal values of serum biomarkers creatinine and potassium in comparison to noninfected animals (Fig. 4A to F).

Gene expression analysis of lung tissue from ExoU^+^ strain 6077-infected rabbits revealed that MEDI3902 promotes significant downregulation of a number of transcripts encoding proinflammatory mediators (IL-1α, IL-1β, IL-6, IL-8, IL-23A, MIP-1α, MIP-1β, MIP-2, MCP-1, GM-CSF, oncostatin M, and LIF) that are important in host immune activation from pathogen recognition, endothelial activation, and neutrophil recruitment to the lung (30–40). In previous studies using P. aeruginosa infection models, blocking the actions of some of these markers (e.g., MCP-1, MIP-1β, and IL-1β) was linked to a beneficial outcome for the resolution of infection, with greater bacterial clearance and diminished tissue damage (41, 42). The downregulation of IL-10 in MEDI3902-treated animals may portend the overall decreased proinflammatory cascade compared to control IgG-treated animals (Table 1). Our data may predict the anti-inflammatory compensatory response is subsequent to the early proinflammatory peak of cytokines, such as IL-6 (43), suggesting that an attenuated inflammatory storm is likely to be followed by an attenuated compensatory response. Importantly, the rise of serum IL-10 along with IL-6 and IL-8 is part of a combined proposed predictor score for fatal outcomes in human sepsis (44), and MEDI3902 administration in the present study resulted in downregulation of all three transcripts (Table 1). Overall, the gene expression attenuation of important proinflammatory markers in MEDI3902-treated animals correlated with greater bacterial clearance and limited tissue-destructing collateral damage derived from uncontrolled immune activation (histopathology analysis). In addition, these effects may be crucial for the avoidance of an uncontrolled release of cytokines (e.g., cytokine storm) that can culminate in hemodynamic disturbances related to sepsis and bacterial spread (45, 46).

In conclusion, we describe the development of a rabbit acute pneumonia model using the highly cytotoxic and pathogenic P. aeruginosa strain 6077 and the testing of bispecific antibody MEDI3902 as our model anti-infective, which has demonstrated strongly protective potential in multiple murine models of infection. We show that this antibody also prevents acute lethal infection in rabbits while also promoting bacterial clearance. These results correlated with blood biomarkers and lung-specific markers of inflammatory cytokine and chemokine expression derived from MEDI3902 and control IgG-treated animals. MEDI3902 is currently under evaluation for the prevention of pneumonia in ventilated subjects colonized with P. aeruginosa (ClinicalTrials.gov identifier https://clinicaltrials.gov/ct2/show/NCT02696902). Whether the results presented herein with MEDI3902 are translatable to clinical success in humans is yet to be determined. However, the consistency of previously reported MEDI3902 efficacy data in mice combined with these additional results in rabbits in which biomarkers can be more closely monitored provide further evidence that MEDI3902 shows promise as a new pathogen-specific anti-infective that might benefit high-risk patients susceptible to P. aeruginosa infection.

## MATERIALS AND METHODS

### Bacterial strain and growth conditions.

P. aeruginosa strain 6077, which is a cytotoxic strain that encodes the type III secreted toxins ExoU, ExoT, and ExoY, was used in the rabbit models. Strain 6077 was a gift from Joanna Goldberg (Emory University). An overnight culture of 6077 was grown in 12 ml of tryptic soy broth (TSB) in a 50-ml vented-cap tube with shaking at 150 rpm and 37°C for 16 to 20 h. The overnight culture (60 μl) was then transferred to 12 ml of fresh TSB and incubated with shaking at 150 rpm and 37°C for 12 h, to an optical density at 600 nm (OD_600_) of approximately 1.8. Bacteria were collected by centrifugation at 16°C, washed once, and then resuspended in lactated Ringer's solution (LRS). The washed cells were then diluted in LRS to an OD_600_ of 1.455 to 1.465, corresponding to 1.2 × 10^9^ CFU/ml. This stock solution of bacteria was further diluted in LRS to a concentration of 9 × 10^7^ CFU/ml for the rabbit acute pneumonia model. The number of bacteria in the inoculum was confirmed by serial dilution on 5% sheep blood agar plates.

### Animal investigation protocol.

The rabbit acute pneumonia model was reviewed and approved by the University of California San Francisco Institutional Animal Care and Use Committee and conducted in a facility accredited by the Association for Assessment and Accreditation of Laboratory Animal Care International. Pathogen-free male New Zealand White outbred rabbits (8 to 12 weeks old, 2.0 to 2.8 kg; Western Oregon Rabbit Co.) were used in all animal studies. Rabbits were housed in single stainless-steel cages in a climate-controlled housing room with a daily 12-h light and 12-h dark cycle. They were provided rabbit food pellets and water *ad libitum*, which were supplemented twice daily with hay and grass and fresh fruits and vegetables (bananas, apples, lettuce, celery, and carrots).

### Rabbit acute pneumonia model.

Rabbits were anesthetized by intramuscular administration of 36 mg/kg ketamine and 5.2 mg/kg xylazine. To induce pneumonia, a 1.5-ml instillation containing 9 × 10^7^ CFU/ml of P. aeruginosa strain 6077 was delivered directly into the lungs of anesthetized rabbits through a 2.5-mm pediatric endotracheal tube that was then immediately removed after instillation of the bacterial inoculum.

For efficacy studies, rabbits (*n* = 6 for each of the experimental groups) were randomized for prophylaxis with intravenous administration through the marginal ear vein of either 15 mg/kg of isotype-matched control IgG (c-IgG) or different doses of MEDI3902 (0.3, 1, 5, or 15 mg/kg). After endobronchial bacterial challenge, these rabbits were assessed every 2 h for the first 36 h postinfection and then three times daily thereafter. Animals with signs of pulmonary dysfunction (respiration rate >75 breaths/minute, cyanosis, and cough) were euthanized for humane reasons with intravenous administration of a lethal overdose of pentobarbital and scored as nonsurvivors; those that survived to the end of the study at 96 h postinfection were euthanized. The investigators were not blinded to the experimental groups.

Two studies were performed for the analysis of biomarkers. In the first study, rabbits (*n* = 6 for each of the experimental groups) were randomized for prophylaxis at 24 h before infection with 15 mg/kg c-IgG or 15 mg/kg MEDI3902 and then euthanized at 10 h postinfection. In the second study, rabbits (*n* = 6 for each of the experimental groups) were randomized for treatment at 1 h postinfection with 15 mg/kg c-IgG or 15 mg/kg MEDI3902 and then euthanized at 10 h postinfection. Two additional rabbits were challenged with lactated Ringer's solution and then euthanized 10 h later and included as vehicle-instilled uninfected controls. In all studies, lungs, spleen, and kidneys were removed aseptically from nonsurvivors and survivors. Lungs, spleens, and kidneys were cut into <0.5-cm pieces, and 0.2- to 0.3-g samples were homogenized in 0.9% saline, followed by quantification of CFU by serial dilutions on 5% sheep blood agar.

### Arterial blood gas analysis.

For analysis of serum biomarkers, arterial blood was collected at 0, 6, and 10 h postinfection and characterized using the Element POC rapid blood analyzer (Heska) for analysis of blood gas (pO_2_, pCO_2_, and base excess), electrolyte (Na^+^, K^+^, Cl^−^, and ionized calcium [iCa^2+^]), and chemistry (creatinine, glucose, and lactate) parameters.

### Lung inflammatory cytokine expression analysis.

Right lungs were harvested, cut into <0.5-cm pieces, and preserved in RNA*later* (Thermo Fisher Scientific) immediately after euthanasia. RNA was extracted using the DNase treatment option with the RNeasy midi kit (Qiagen), followed by cDNA synthesis according to the manufacturer's instructions. Gene expression profiles were then evaluated using the rabbit Inflammatory Cytokines & Receptors Real-Time Reverse Transcriptase (RT^2^)-Profiler kit (Qiagen), according to the manufacturer's instructions. Each RT^2^ Profiler was performed twice per animal, which included evaluation of a technical replicate. The RNA integrity number (RIN) evaluation (>5), PCR array reproducibility, reverse transcriptase efficiency, and genomic DNA contamination were evaluated for each RNA extraction for quality control purposes. Average cycle threshold (*C_T_*) values were used for each tissue, with a threshold of 0.051. The mean *C_T_* values of four rabbit housekeeping genes (HKGs), ACTB, GAPDH, LDHA, and loc100346936, were used for normalization. For each gene, the fold regulation was evaluated as fold change of the MEDI3902 treatment group relative to the c-IgG treatment group using normalized *C_T_* values.

### Histology.

The left lungs were inflated by gravity with 10% neutral buffered formalin, fixed for 72 h at 8°C, and then transferred to 70% ethanol. Fixed tissues were processed according to standard methods, as described previously (47), and stained with Gill's hematoxylin (Mercedes Medical, Sarasota, FL) and eosin (Surgipath, Richmond, IL) for histologic evaluation by a pathologist blinded to the experimental conditions.

### Statistical analysis.

The predetermined sample size of 12 rabbits per experimental group was estimated using log rank test with 5% type I error rate and 80% power. However, a preplanned interim analysis that was conducted after data were collected for 6 rabbits per experimental group for the initial efficacy study revealed a particularly potent dose-dependent protective effect of MEDI3902 in preventing death in this model. As such, the study was terminated earlier than planned and included data from only 6 rabbits per experimental group. Accordingly, the sample size of 6 rabbits per experimental group was subsequently used for the biomarker studies. Survival curves were generated using the Kaplan-Meier method, and significance was assessed by means of the log rank (Mantel-Cox) test. Normal distribution was not assumed, so variables were compared using a nonparametric two-sided Mann-Whitney U test.

For analysis of RT-PCR data, a one-way analysis of variance (ANOVA) model with heterogeneous within group variance was applied for MAb prophylaxis and treatment studies indicating no difference between the control IgG groups (*P* > 0.05). This allowed for an evaluation of the gene fold regulation of MEDI3902-treated animals relative to the pooled control IgG animals. The 95% confidence interval for fold regulation and the false-discovery rate (FDR) adjusted *P* values are reported (48). Only genes with similar melting temperature values within replicates were evaluated.
